# Effort, reward and self-reported mental health: a simulation study on negative affectivity bias

**DOI:** 10.1186/1471-2288-11-121

**Published:** 2011-08-24

**Authors:** Marc Arial, Pascal Wild

**Affiliations:** 1 Institute for Work and Health, Lausanne University and Geneva University, Bugnon 21, CH-1011 Lausanne, Switzerland; 2 PW Statistical Consulting, 56, avenue Paul Déroulède, 54520 LAXOU- France

## Abstract

**Background:**

In the present article, we propose an alternative method for dealing with negative affectivity (NA) biases in research, while investigating the association between a deleterious psychosocial environment at work and poor mental health. First, we investigated how strong NA must be to cause an observed correlation between the independent and dependent variables. Second, we subjectively assessed whether NA can have a large enough impact on a large enough number of subjects to invalidate the observed correlations between dependent and independent variables.

**Methods:**

We simulated 10,000 populations of 300 subjects each, using the marginal distribution of workers in an actual population that had answered the Siegrist's questionnaire on effort and reward imbalance (ERI) and the General Health Questionnaire (GHQ).

**Results:**

The results of the present study suggested that simulated NA has a minimal effect on the mean scores for effort and reward. However, the correlations between the effort and reward imbalance (ERI) ratio and the GHQ score might be important, even in simulated populations with a limited NA.

**Conclusions:**

When investigating the relationship between the ERI ratio and the GHQ score, we suggest the following rules for the interpretation of the results: correlations with an explained variance of 5% and below should be considered with caution; correlations with an explained variance between 5% and 10% may result from NA, although this effect does not seem likely; and correlations with an explained variance of 10% and above are not likely to be the result of NA biases.

## Background

Assessing the effect of a deleterious psychosocial work environment on mental health has emerged in recent years as a major scientific challenge. Mental disorders are an important health issue in most countries [1]. Co-morbidities, frequent and chronic illnesses and disabilities have thus also become major concerns for many organizations. Scientific evidence suggests that the psychosocial environment at work may influence the mental well-being of an organization's employees. The Effort Reward Imbalance (ERI) model [2] is a theoretical approach for conceptualizing a deleterious psychosocial work environment. The idea behind this model is that social reciprocity is a major principle of the work contract and that failed reciprocity (resulting from high perceived efforts and low perceived rewards) causes workers to experience stress related strain [3]. This model predicts several adverse health outcomes, such as poor self-reported health [4], cardiovascular problems [5] and musculoskeletal disorders [6]. Several studies also suggest that an imbalance between effort and reward in the workplace may be associated with poor mental health [7-10].

Questionnaire-based methods in research on occupational health are broadly accepted in the scientific community. Typical studies that use these methods include self-reported measures of exposure to risk factors and self-reported measures of health (or of some specific component of a participant's health or symptoms). However, some authors suggest that using the same method to measure the independent variables and the dependent variable may lead to inflated correlations. Furthermore, scientific evidence suggests that those inflated correlations may result from diverse biases [11] such as Negative Affectivity (NA). NA is a personality trait that predisposes a person to experience negative emotions and that causes a general pessimistic view of the world. Thus, it is expected that persons with NA will be more likely to report that their job is stressful, demanding and dissatisfying. These persons are also expected to perceive their health problems and symptoms as more serious than they would if they did not have NA. Research regarding the effect of occupational stressors on the mental health of workers indicates that NA may be a factor in this effect, as NA has the potential to inflate correlations between independent (e.g., work characteristics) and dependent (e.g., mental health) variables, leading to erroneous conclusions.

Researchers have employed a variety of strategies to control for NA-related biases. Most of these strategies are based on prospective study designs or on adjustments for NA in multiple regression models. However, both of these types of strategies (and their combinations) are known to have several limitations. In the present article, we propose an alternative way to manage NA in research when investigating the relationship between a deleterious psychosocial environment at work and poor mental health. A general approach to causal inferences was suggested that includes [12] establishing the existence of a relationship between variables and ruling out plausible alternatives. Our approach to studying NA issues in the relationship between the psychosocial environment at work and poor mental health consisted of the following: first, we determined how strong NA has to be to cause a correlation between the independent and dependent variables; second, we subjectively assessed whether NA may be important enough and widespread enough among our subjects to invalidate the results of our research.

## Methods

We based our assessment of the influence of NA on a simulation study. Specifically, we investigated the effect of effort reward imbalance [2] on mental health.

Efforts and rewards were measured by 6 and 11 Likert-scaled items, respectively. The effort scale includes items about workload/time pressure, interruptions and responsibility, for example. The reward scale consists of three subscales: esteem (5 items); job promotion (4 items); job security (2 items). A complete list of items can be found in the appendix [13]. The rating procedure for all items included in the effort and reward scales is as follows: 1- does not apply; 2- does apply but the respondent does not consider himself/herself distressed; 3- does apply and the respondent considers himself/herself somewhat distressed; 4- does apply and the respondent considers himself/herself distressed; and 5- does apply and the respondent considers himself/herself very distressed. Different formulations for the effort and reward imbalance are suggested in the scientific literature. Using the data from the GAZEL cohort, [14] concluded that the continuous ratio and the log transformed ratio were the most powerful formulations among those they investigated. We performed our analysis for both those formulations in order to verify whether they also made a difference in terms of NA sensitivity. The continuous ratio was calculated by dividing the score for effort by the score for reward. A correction factor was applied to the score for reward in order to adjust for the unequal number of items. The log transformed ratio results from the logarithmic transformation of the continuous ratio [15]. The logic behind this transformation is to place inverse imbalance of the same magnitude in the same distance from 1. [14] also suggested to explore the separate effects of effort and reward. This might be relevant if only one of those components could be significant in the population studied. Therefore, we also performed our analysis using the scores for effort and reward separately in a multiple linear regression.

We used the General Health Questionnaire (GHQ) to measure poor mental health [16]. This questionnaire asks whether the respondent recently experienced any specific symptoms or behaviors. The scale has been shown to be a valid first-stage screening instrument for current diagnosable psychiatric disorders [17]. Many studies have assessed the psychometric characteristics of the GHQ. For example, some studies have shown values between 0.75 and 0.90 for test-retest reliability [18]. Other studies also concluded that this scale has an internal consistency estimate (Cronbach's alpha) ranging from 0.81 to 0.86 [19-21]. We used the French version of the scale, which has been validated in different populations [22,23]. We used the 12-item version [17] of the questionnaire (GHQ-12). This version is the shortest and most commonly-used version of the GHQ [24]. We used the GHQ Likert score as a scoring method [25]. Possible values ranged from 0 to 36. The score was evaluated as a continuous variable in our regression analyses.

We simulated 10,000 populations of 300 subjects each. Each item on the two questionnaires was simulated independently, according to the observed marginal distribution in an actual population of workers. Thus, we postulated that no relationship existed between mental health and job stressors. The dataset we used as a basis for our simulation originates from mental health study of out-of-hospital care providers [26]. This study took place in the French speaking part of Switzerland in 2009. 333 out-of-hospital care providers (258 men, 75 women; mean age = 34.8, SD = 7.5) answered a questionnaire including questions on mental health (GHQ-12), demographics, health-related issues and work characteristics, questions from the ERI Questionnaire, and items about overcommitment. The effort-reward ratio appeared associated to poor mental health (P < 0.001) in a two-level multiple regression. Other variables included in the model were: overcommitment score; weekly number of interventions; percentage of non-prehospital transport of patients out of total missions; gender; and age.

To assess the influence of NA in the present simulation study, we simulated NA in each of the populations in varying proportions (5%, 10% and 20% of the subjects) and varying intensities (10%, 20%, 30% and 40% of the items were affected). Those percentages were arbitrarily chosen. The underlying principle was to obtain a broad spectrum of values for the percentage of populations for which a significant association was observed (values ranged from 5% to 100%, Table 1), while choosing NA influence in the range of what we assumed to be plausible. The NA of each subject was sampled according to the assumed proportion - for example, if the assumed proportion was 10%, each subject had a 10% probability of displaying NA. For each subject that was identified and sampled as having NA, we sampled a given proportion of the items that were influenced by NA, which varied from 10% to 40% of the items. When an item for a specific subject was identified as being influenced by NA, we ascribed that item with the next "negative" value to its original value. This procedure was applied equally to each item from the Siegrist's questionnaire and the GHQ-12.

**Table 1:** Proportion of explained variance (R2) and proportion of significant coefficients (p-values below 5%) in the relationship between the log GHQ-12 Likert score with the log-transformed ERI ratio, ERI ratio, and effort and reward score by the degree of NA influence across 10,000 simulated populations

		Items affected by NA									
		**0%**		**10%**		**20%**		**30%**		**40%**	

	**subjects with NA**	**R2**	**Proportion of significant coef**.	**R2**	**Proportion of significant coef**.	**R2**	**Proportion of significant coef**.	**R2**	**Proportion of significant coef**.	**R2**	**Proportion of significant coef**.

Model ^1 ^including the log-transformed ERI	5%	0.34%	0.05	0.36%	0.06	0.50%	0.11	1.00%	0.29	2.00%	0.59
	
	10%	0.34%	0.05	0.39%	0.07	0.85%	0.23	2.33%	0.68	5.08%	0.95
	
	20%	0.34%	0.05	0.46%	0.09	1.73%	0.54	5.34%	0.98	11.28%	1.00

Model ^1 ^including the ERI score	5%	0.34%	0.05	0.36%	0.06	0.52%	0.12	1.13%	0.33	2.41%	0.67
	
	10%	0.34%	0.05	0.39%	0.07	0.92%	0.26	2.65%	0.74	5.95%	0.98
	
	20%	0.34%	0.05	0.46%	0.09	1.86%	0.57	5.86%	0.98	12.47%	1.00

Model ^2 ^including the effort and reward			e^a ^/r^b ^/er^c^		e^a ^/r^b ^/er^c^		e^a ^/r^b ^/er^c^		e^a ^/r^b ^/er^c^		e^a ^/r^b ^/er^c^
	
	5%	0.68%	0.05/0.05/0.00	0.70%	0.05/0.05/0.00	0.88%	0.08/0.08/0.01	1.51%	0.17/0.15/0.04	2.77%	0.27/0.21/0.16
	
	10%	0.68%	0.05/0.05/0.00	0.73%	0.06/0.06/0.00	1.31%	0.14/0.13/0.03	3.12%	0.28/0.23/0.21	6.42%	0.21/0.15/0.61
	
	20%	0.68%	0.05/0.05/0.00	0.82%	0.07/0.07/0.01	2.37%	0.26/0.21/0.11	6.62%	0.19/0.13/0.65	13.41%	0.03/0.01/0.96

^a ^Effort is significant and Reward is not significant

^b ^Reward is significant and Effort is not significant

^c ^Effort and Reward are significant

^1 ^Model based on simple linear regression

^2 ^Model based on multiple linear regression

In order to assess the extent to which NA can induce spurious associations between stressors at work and mental health, we performed three linear regressions, each with the GHQ-12 score as the dependent variable. The first two were regression procedures with a single independent variable: one using the untransformed effort-reward ratio and the other using the log-transformed effort-reward ratio. In the third regression we fitted the reward and the effort scores as two independent variables.

For each of the computed models we stored the R2 statistic and whether or not each of the coefficients corresponding to the independent variable(s) were statistically significant at the 5% level.

We summarized the effect of our simulated NA by computing the mean R2s across our 10,000 simulated populations and the proportion of significant coefficients. The latter is of course also dependent on the size of each population (in our case n = 300) but as statistical significance is the usual criterion by which an effect is bound to occur, we found it interesting to present this statistic.

## Results and discussion

Table 2 shows the mean scores (SD) of the log of the GHQ Likert score, the effort score, the reward score and the ERI ratio by the degree of NA influence across the 10,000 simulated populations. Table 1 shows the proportions of the explained variance (R2) and the proportions of p-values below 5% in the relationship between the log of the GHQ Likert score with the log-transformed ERI ratio, the ERI ratio, and the effort and reward score by the degree of NA influence across the 10,000 simulated populations.

**Table 2:** Mean scores (SD) of the log GHQ-12 Likert score, effort score, reward score and ERI ratio by the degree of NA influence across 10,000 simulated populations

		Percentage of items affected by NA				
**Mean scores (SD) across 10,000 simulated populations**	**Percentage of subjects with NA**	**0%**	**10%**	**20%**	**30%**	**40%**

Log GHQ12 Likert Score ^a^	5	2.29(0.20)	2.29(0.21)	2.30(0.21)	2.30(0.21)	2.31(0.22)
	
	10	2.29(0.20)	2.30(0.21)	2.31(0.21)	2.32(0.22)	2.32(0.23)
	
	20	2.29(0.20)	2.31(0.21)	2.33(0.22)	2.35(0.24)	2.36(0.25)

Effort score (Siegrist) ^b^	5	12.5(1.9)	12.5(1.9)	12.5(1.9)	12.6(2.0)	12.6(2.0)
	
	10	12.5(1.9)	12.5(1.9)	12.6(2.0)	12.6(2.0)	12.7(2.1)
	
	20	12.5(1.9)	12.6(1.9)	12.7(2.0)	12.8(2.1)	12.9(2.2)

Reward score (Siegrist) ^c^	5	48.1(3.2)	48.0(3.3)	48.0(3.3)	47.9(3.3)	47.8(3.4)
	
	10	48.1(3.2)	48.0(3.3)	47.8(3.3)	47.7(3.4)	47.6(3.5)
	
	20	48.1(3.2)	47.8(3.3)	47.6(3.4)	47.4(3.5)	47.2(3.7)

Effort-reward Imbalance ratio (Siegrist)	5	0.478(0.081)	0.480(0.081)	0.481(0.083)	0.483(0.085)	0.485(0.088)
	
	10	0.478(0.081)	0.481(0.082)	0.485(0.085)	0.489(0.089)	0.493(0.095)
	
	20	0.478(0.081)	0.485(0.084)	0.492(0.089)	0.500(0.096)	0.508(0.105)

^a ^range possible values: 0 to 36

^b ^range possible values: 6 to 30

^c ^range possible values: 11 to 55

Note: the unequal number of digits is due to the fact that variables considered have different scales.

Table 1 shows that increasing the number of subjects and items affected by NA also increases the number of simulated populations for which a significant correlation is identified. Those results can be compared to observed correlations to assess whether the observed association is a true association or whether the relationship results from NA in some subjects. For example, if we observe a significant correlation between the ERI ratio and the GHQ-12 score in the reference population and an explained variance of 6%, according to the results of our simulation study, these parameters would be interpreted in the following manner: for NA to be the unique variable that causes the observed association, it would need to affect at least 20% of the subjects in 30% (average) of the items or 10% of the subjects in 40% of the items. The next step in this analysis would be to subjectively assess the plausibility of the following assertion: is it plausible that so many subjects in the reference population have the NA trait? Considering the items used, is it realistic to expect that 30% of the answers given by those subjects are biased? Our procedure does not clearly exclude or confirm the role of NA in an observed association, but the procedure suggests how large NA's effect has to be to invalidate an observed association. Our results show that NA has to be important and widespread among subjects in order to create spurious correlation between ER ratio and poor mental health. This suggests that the biasing effect of NA alone is unlikely to create the correlations observed in many studies between ERI and poor mental health. This conclusion is also coherent with findings from prospective studies [3,8,10].

Based on the results from Table 1 we would suggest the following general rules when investigating and interpreting the results regarding the association between the ERI ratio and the GHQ-12 score: correlations with an explained variance of 5% and below should be considered with caution; correlations with an explained variance between 5% and 10% may result from NA, although this effect is unlikely; and correlations with an explained variance of 10% and above are not likely to result from NA biases but from additional factors.

The results of the present study show that simulated NA has a minimal effect on the mean scores for effort and reward. The differences that were observed between the simulated populations without NA and those with high levels of NA are insignificant when compared to the inter-population differences that are referenced in the scientific literature. For example, an article that discussed the results of 5 major European studies researching the ERI in diverse populations [13] reported mean scores ranging from 8.2(2.8) to 14.4(2.5) for effort and from 28.9(4.8) to 46.6(8.2) for reward. This does not exclude potential inflated correlations between ERI and GHQ scores. However, our results further support the well-accepted theory that inter-population differences in the average scores for effort and reward are not solely the consequence of differences in NA in those populations. In other words, although this scale includes strong perceptual components, ERI does not appear to be a proxy of NA.

A modest effect of NA on mean scores for dependent and independent variables (Table 2) does not always indicate an inconsequential effect of NA on the correlations that are observed between those variables (Table 1).

As mentioned in the introduction, many strategies have been used to deal with potential biasing effect of NA. Several of these strategies are based on multiple regressions and involve adjusting for NA by including an array of variables that reflect a subject's tendency to perceive the world in a negative way. For example, [27] included in their multivariable model a scale calculated from a broad range of items measuring the reported level of annoyance due to various environmental factors, such as heat, cold, dust and light. Similarly,[14] adjusted for those scales that involved strong personality components (e.g., Siegrist's over-commitment scale, scales for depressive disorders) to avoid potential NA biases. However, as pointed out by Spector and colleagues [28], in every measure of NA, there is one part of the measure that functions as a strain measure that can be influenced by work stressors. It is therefore impossible to differentiate the NA characteristic from its strain measure in cross-sectional questionnaire studies. In this seminal paper, the authors strongly advised against adjusting for NA measures when analyzing correlations between self-reported work stressors and measures of health. Our simulation procedure offers an alternative to adjusting for NA in multiple regression. We believe that our procedure allows for the subjective assessment of the validity of observed correlations without removing the substantive effect of the variables considered.

Our study has some limitations worth noting. First, we postulated that NA has the same potential influence on all of the items on the questionnaire. This postulate simplifies the simulation but it does not reflect the real sensitivity to NA of the items from the GHQ-12 or from the Siegrist questionnaire. Second, the models shown in Table 1 explain very little variance. This results from our simulation procedure: the underlying principle here was to "remove" the actual association observed in the original dataset in order to have no correlation between ERI and GHQ-12 scores. In doing so, we removed the coherence from the data and only limited variance remained (resulting from error and simulated NA). Third, we based our simulations on the marginal distribution of items from the Siegrist's questionnaire and the GHQ-12 in an actual population of workers. Compared to previous studies, this population of workers had a relatively low proportion of cases with high effort and low reward. However, we observed a correlation between high effort, low reward and the GHQ score; therefore, we assert that this low proportion of cases with high effort and low reward should not be a major issue regarding the validity of our conclusions for the present simulation study. We further conclude that applying a similar procedure and using the same questionnaires in different populations of workers may strengthen the validity of our findings.

## Conclusions

The simulation procedure suggested in this article does not replace rigorous study design or objective measurement tools. Using prospective study designs to investigate the potential relationships between the independent and dependent variables that are probably influenced by NA is an effective way to avoid inflated correlations that are due to NA. Using objective measures for stressors and health outcomes, or using methods that objectivate those measures (e.g., multilevel/hierarchical modeling), are promising ways to control the potential biasing effect of NA. The simulation procedure that we applied in the present study could be used as a complement to the usual methods used to address those NA issues in research on stressors and their consequences on the mental health of workers.

## List of abbreviations

ERI: Effort Reward Imbalance; GHQ: General Health Questionnaire; NA: Negative Affectivity

## Competing interests

The authors declare that they have no competing interests.

## Authors' contributions to the study

MA and PW jointly conceived the main idea behind the article. PW analyzed the data. MA and PW drafted the manuscript. All authors read and approved the final manuscript.

## Pre-publication history

The pre-publication history for this paper can be accessed here:

http://www.biomedcentral.com/1471-2288/11/121/prepub
